# Liver transplantation for acute liver failure due to antitubercular drugs – a single-center experience

**DOI:** 10.6061/clinics/2018/e344

**Published:** 2018-06-23

**Authors:** Rodrigo Bronze de Martino, Edson Abdala, Felipe Castro Villegas, Luiz Augusto Carneiro D’Albuquerque, Alice Tung Wan Song

**Affiliations:** I Disciplina de Transplante de Figado e Orgaos do Aparelho Digestivo, Departamento de Gastroenterologia, Hospital das Clinicas HCFMUSP, Faculdade de Medicina, Universidade de Sao Paulo, Sao Paulo, SP, BR; II Laboratorio de Hepatite Virais – LIM-47, Faculdade de Medicina, Universidade de Sao Paulo, Sao Paulo, SP, BR; III Departamento de Doencas Infecciosas, Hospital das Clinicas HCFMUSP, Faculdade de Medicina, Universidade de Sao Paulo, Sao Paulo, SP, BR; IV Laboratorio de Transplante e Cirurgia de Figado - LIM-37, Faculdade de Medicina, Universidade de Sao Paulo, Sao Paulo, SP, BR

**Keywords:** Tuberculosis, Hepatic Insufficiency, Drug Toxicity, Survival, Liver Transplantation

## Abstract

**OBJECTIVES:**

Patients receiving treatment for tuberculosis are at risk of developing acute liver failure due to the hepatotoxicity of antitubercular drugs. We aimed to describe our experience with liver transplantation from deceased donors in this situation.

**METHODS:**

We identified patients undergoing transplantation for acute liver failure due to antitubercular drugs in our prospectively maintained database.

**RESULTS:**

Of 81 patients undergoing transplantation for acute liver failure, 8 cases were attributed to antitubercular drugs during the period of 2006-2016. Regarding the time of tuberculosis treatment until the onset of jaundice, patients were on antitubercular drugs for a mean of 64.7 days (21-155 days). The model for end-stage liver disease (MELD) score of patients ranged from 32 to 47 (median 38), and seven patients underwent transplantation under vasopressors. The 1-year survival was 50%. Three patients died during the week following transplantation due to septic shock (including a patient with acute liver failure due to hepatic/disseminated tuberculosis), and the remaining patient died 2 months after transplantation due to pulmonary infection. There were 2 cases of mild rejection and 1 case of moderate rejection. Of the surviving patients, all were considered cured of tuberculosis after alternative drugs were given.

**CONCLUSION:**

Patients arrived very sick and displayed poor survival after deceased donor transplantation.

## INTRODUCTION

Tuberculosis is one of the top 10 causes of death worldwide, despite being a curable infectious disease 1. Brazil is one of the countries with a high burden of tuberculosis, with an estimated incidence of 44/100,000 pulmonary tuberculosis in 2014. Because Brazil is a large country, the incidence of tuberculosis varies according to the area, city size, population density and socioeconomic indicators 2.

The cornerstone of tuberculosis treatment consists of a regimen containing rifampicin, isoniazid, pyrazinamide and ethambutol. The first three drugs are potentially hepatotoxic 3, and the incidence of hepatoxicity has been reported to vary between 2% and 28% 4. More often, these drugs lead to an asymptomatic increase in serum aminotransferase levels, but acute liver failure (ALF) even after the discontinuation of the drugs has also been reported 5. Antitubercular drug-induced hepatotoxicity is related to an idiosyncratic reaction to the released metabolites and can occur independently of the dose 4,6.

ALF is a life-threatening condition with variable mortality rates, depending on etiology and age, among other factors 7. Liver transplantation (LT) is the only treatment for cases within previously established criteria 8. In Europe, 8% of LT are due to ALF, and 18% of these cases are due to drug-induced liver damage 7. LT for ALF due to antitubercular drugs presents peculiarities for management and outcome prediction that deserve to be better understood. Some published case reports and series have described varying survival rates after LT for ALF. Moreover, patients in this context may present with a confounding symptom, as the disease itself can present as diffuse hepatic infiltration, also known as granulomatous hepatitis leading to ALF 9.

In this study, we aimed to describe our experience with LT for ALF resulting from the use of antitubercular drugs to aid in the decision-making for LT in patients in this situation.

## METHODS

For case identification, we initially identified all patients listed for ALF in our informatized national list over a 10-year period (2006-2016). Afterwards, we consulted our prospectively maintained database to identify cases in which drug toxicity was the cause of ALF and finally consulted informatized patient charts to identify all patients undergoing LT for ALF related to tuberculosis treatment.

### ALF management protocol

Patients referred to our unit with ALF are systematically subjected to the following protocol: laboratory workup including serologies for viral hepatitis, HIV, HTLV, toxoplasmosis, cytomegalovirus, syphilis, Chagas, HCV RNA quantification, autoimmune autoantibodies, factor V, and ABO typing, in addition to routine hematological and biochemical exams; radiological workup includes Doppler ultrasound of the upper abdomen; procedures such as central line and intracerebral monitoring are performed if indicated; and written consent is obtained from the family for listing and transplantation. Antimicrobial treatment includes cefotaxime and fluconazole. Post-transplantation prophylaxis includes ivermectin, sulfametoxazol-trimetoprim, and preemptive approach for cytomegalovirus.

Patients who met the previously established criteria (King’s College or Clichy) were listed for LT 10,11.

The immunosuppression protocol consisted of 500 mg intraoperative methylprednisolone, tapering until 20 mg prednisone was reached, followed by weaning over 3 months. Tacrolimus was initiated on the first post-operative day aiming at a serum level of 8-10 ng/ml. Sodium mycophenolate was added if there was renal insufficiency, and in this context, the target serum level of tacrolimus was 5-8 ng/ml.

## RESULTS

From 2006 to 2016, 81 patients underwent LT for ALF at our institution. Fourteen (17.2%) cases were due to drug toxicity, and of these, 8 were related to antitubercular drugs.

All eight patients were women, with a median age of 39 years (range 17-56). None of the patients had any history of liver disease; three had hypertension, and one had hypothyroidism as comorbidities.

### Clinical and laboratory data

Six patients presented with the acute form of liver failure, and two patients presented the hyperacute form according to previous classifications 12. The interval between jaundice and encephalopathy onset ranged from 2 to 27 days (mean 13.1 days). Regarding the grade of encephalopathy, 4 patients were transplanted with West Haven grade II encephalopathy 13, 2 had grade III, and 2 had grade IV encephalopathy. On admission, the median liver functional values were as follows: AST 459 U/L (167-1623 U/L); ALT 555 U/L (141-265 U/L); TB 22.9 mg/dL (6.21-44.2 mg/dL); INR 4.34 (1.7-10.7); creatinine 0.72 mg/dL (0.42-1.74 mg/dL); model for end-stage liver disease (MELD) score 34 (30-40); and factor V 25% (13-31%).

The median MELD score immediately before transplantation was 38 (32-47). The median laboratory values immediately before transplantation were TB 23.2 mg/dL (11.4-39.3); INR 3.33 (1.29-5.09); Creatinine 1.73 mg/dL (0.32-3.65 mg/dL); and factor V 25% (15-36%).

After being listed as priority patients, the waiting time until transplantation ranged from a few hours to 17 days (median 4 days).

All eight patients had severe hepatic insufficiency, and five patients underwent transplantation under mechanical ventilation. Two patients required renal replacement therapy before transplantation, and all of them did so after transplantation. Six patients required vasopressors before transplantation.

Of the 8 transplanted patients, 4 had an additional active infection at the time of transplantation, all of pulmonary site (2 unknown etiology, 1 carbapenem-resistant *Acinetobacter baumanii* and 1 ESBL-producing *Klebsiella pneumoniae*).

### Pre- and post-transplantation tuberculosis data

Six patients had pulmonary tuberculosis; the remaining two had ocular and bone tuberculosis. One patient diagnosed with pulmonary tuberculosis was later diagnosed as having disseminated disease that had infiltrated the lung, liver and bowels. The diagnosis of tuberculosis was done by positive direct bacilloscopy in four patients, positive culture in two, *Mycobacterium tuberculosis* PCR in one, and histological, clinical and radiological characteristics in the remaining case (Table 1). Regarding the time of tuberculosis treatment until the onset of jaundice, patients were on antitubercular drugs for a mean of 69 days (21-155 days) and a median of 34 days. Four of the 8 transplanted patients received at least 2 months of antitubercular treatment before transplantation. All patients were using rifampicin and isoniazid; seven were on pyrazinamide; and six were using ethambutol at the beginning of the symptoms.

After transplantation, patients received an alternative treatment for tuberculosis based on ethambutol in 6 cases, levofloxacin in 6 cases, streptomycin in 2 cases, ciprofloxacin in 1 case, azithromycin in 1 case, and linezolid in 1 case. The 4 patients who were alive at one year after transplantation had received alternative tuberculosis treatment for 6 months (1 patient), 9 months (2 patients) and 1 year (1 patient). All were considered cured, with no symptoms suggestive of recurrent tuberculosis.

### Outcome

The 1-year survival rate after the transplantation was 50%; two deaths occurred at 1 and 7 days post-transplantation and were related to sepsis and multiorgan failure after severe hemodynamic instability; one patient died 2 months after the procedure due to sepsis from pulmonary infection; and the last patient presented with hepatomegaly on admission and underwent LT for ALF due to antitubercular drugs after 1 month of treatment. Liver pathology showed a 1540-g liver with granulomas with caseum. He died 2 days after the transplantation due to disseminated disease that had infiltrated the lung, liver and bowels. All 4 patients who died were on vasopressor drugs pre-transplantation; 1 patient had grade II encephalopathy, and the others had grade III or IV encephalopathy, with a MELD score ranging from 33 to 47. Of the 4 patients with 1-year survival, only 2 were on vasopressors at the time of transplantation; 3 had grade II encephalopathy, and 1 had grade IV encephalopathy, with a MELD score ranging from 32 to 42. The surviving patients were followed up for a mean of 1991 days.

### Pathology

On liver pathology, organ weight varied from 463.9 g to 1540 g. Histology showed massive and sub-massive hepatic necrosis in all cases, but in one patient, the necrosis was present in a micronodular liver cirrhosis background. In this case and another one, granuloma with caseum was found.

## DISCUSSION

To the best of our knowledge, this study has the largest number of transplanted patients in a single center for ALF due to antitubercular drugs. Previously published studies include series of cases and isolated case reports 14-17, and our series may contribute to the current literature regarding patient characteristics before transplantation and management of tuberculosis and outcome after transplantation.

As ours is a national reference center, 7 out of the 8 patients were referred in critical condition from other hospitals. More than half of the patients underwent transplantation under both ventilatory and circulatory support, which could explain the low 1-year survival rate of 50% even for LT due to ALF, which is typically worse than LT for chronic liver disease 18,25. Compared to the patients who had favorable outcomes, the patients who had poor outcomes underwent surgery on vasopressor drugs (50 *vs* 100%) and had grade III or IV encephalopathy (1 *vs* 3). Two of those patients with worse outcomes, in fact, had bad indications for the procedure. One had disseminated tuberculosis, and the other probably had an acute-on-chronic liver disease triggered by antitubercular drugs. The survival rate in our report is equivalent to the one described by Ichai et al. (50%), which was, until recently, the study with the largest number of transplanted patients in the same context in a single center 14. Patients with ALF are often in critical conditions, and some of them are very young. There are no current well-established factors that can predict the outcomes after transplantation. Despite the effort of some authors, this issue remains to be better understood 19,20, and as some authors suggest, the critical decision for transplantation should be made on a case-by-case basis 21.

A recent comprehensive review reported on 19 transplanted individuals without known liver disease with good long-term survival rates – however, 6 of these patients were from the Ichai cohort, and 10 were single case reports 17. Based on these data, we can conclude that in general, single case reports describe cases with favorable outcomes, and authors are usually less motivated to publish cases with negative results, and therefore the reported survival rate in the review may have been overestimated. Although another recently published paper demonstrated excellent survival rates for 6 individuals undergoing emergency living donor transplantation in the same context, little information is given regarding pre-transplantation characteristics of the patients and life support, with a mean MELD score of 30.1 (compared to the mean score of 38 in our cohort). The 6 transplanted individuals came from a pool of 19 patients with ALF, and possibly those with better clinical parameters were given the chance of living donor transplantations, considering the risks for the donor 15. Another recent paper described a good survival rate of 71.4% in 7 patients undergoing living donor transplantations in India, with a median follow-up of 22 months 16. Although the authors did not mention any patient who were unfit for transplantations, patients went to the procedure very sick, with a mean MELD score greater than 35.

Once patients are referred to our clinic under antitubercular drugs, it may be difficult to differentiate whether hepatic damage is due to therapy or related to liver infiltration of disseminated tuberculosis. Additionally, it seems that the prognosis is completely different under these two circumstances. In their series, Ichai et al. 14 also reported one patient who died after the LT due to disseminated tuberculosis, and similar cases are described in single case reports, mostly post-mortem 9,22-24. In the described cases, hepatomegaly may be a signal of a disseminated disease into the liver rather than liver damage caused by drugs.

Treatment after transplantation did not include the possible causative hepatotoxic drug, and alternative regimens were capable of leading to cure in 100% of the surviving cases. Other studies have reported similar findings, despite the recommendation that any mycobacterial infection should be optimally treated with documented microbiologic and radiological resolution before transplantation is considered 26.

One major difficulty in administering antitubercular therapy to transplantation patients is drug-drug interactions involving rifampin 27. This drug and, to a lesser degree, isoniazid induces the metabolism of tacrolimus via the induction of the cytochrome P450 pathway, and thus the incidence of graft rejection may be significant in solid organ transplantation recipients.

There are some limitations to our study. It is a single-center series, with a possible bias of patients with more severe disease undergoing LT. Moreover, the number of cases described is small.

In conclusion, patients were very sick at the time of transplantation and displayed poor survival after deceased donor transplantation. Except for the case in which the patient died of disseminated tuberculosis, fatal outcome was not related to tuberculosis but to complications of the transplantation performed in patients in severe clinical conditions. Current literature is lacking more sensitive diagnostic tools to predict outcomes in ALF that can help avoid futile procedures.

## AUTHOR CONTRIBUTIONS

Martino RB, Abdala E, D’Albuquerque LA and Song AT designed the study, Villegas FC collected the data. Martino RB, Villegas FC and Song AT analyzed the data. Martino RB, Song AT and Abdala E wrote the paper. All authors read and approved the final version of the manuscript.

## Figures and Tables

**Table 1:** Demographic and clinical characteristics of patients undergoing liver transplantation for acute liver failure due to antitubercular drugs.

Patient	Age (years)	Site of infection	Duration of Anti-TB treatment before ALF (days)	Anti-TB treatment pre-LT	Type of ALF	MELD	HE (grade)	Pre-LT VAD	Pre-LT Dialysis	Anti-TB treatment post-LT	Outcome (days)	Rejection
*1*	17	Pulmonary	66	RIPE	Acute	42	IV	+	-	ETH + LFX	Alive (483)	Moderate AR
*2*	17	Disseminated	28	RIPE	Hyperacute	47	III	+	-	SM + LFX	Dead (2)	-
*3*	18	Pulmonary	40	RIPE	Hyperacute	33	III	+	-	AMC + LFX + ETH	Dead (7)	-
*4*	56	Pulmonary	40	RIPE	Acute	38	IV	+	-	LFX + SM + ETH	Dead (62)	Mild AR
*5*	59	Ocular	133	RIF + INH	Acute	32	II	+	+	ETH + LFX	Alive (3311)	-
*6*	38	Pulmonary	58	RIF + INH + PZA	Acute	42	II	-	-	CFX + ETH	Alive (3874)	Mild AR
*7*	56	Bone	174	RIPE	Acute	43	II	+	+	-	Dead (1)	-
*8*	41	Pulmonary	36	RIPE	Acute	32	II	-	-	LFX + LNZ + ETH	Alive (397)	-

ALF, acute liver failure; AMC, azithromycin; AR, acute rejection; CFX, ciprofloxacin; ETH, ethambutol; INH, isoniazid; LFX, levofloxacin; LNZ, linezolid; PZA, pyrazinamide; RIF, rifampicin; RIPE, rifampicin-isoniazid-pyrazinamide-ethambutol; SM, streptomycin; TB, tuberculosis; HE, hepatic encephalopathy; MELD, model for end-stage liver disease; VAD, vasoactive drugs.
